# Supplementary material for: Probabilistic Circuits for Autonomous Learning: A Simulation Study
Source: Front Comput Neurosci. 2020 Feb 25;14:14. doi: 10.3389/fncom.2020.00014 (PMC7052495; doi:10.3389/fncom.2020.00014)
Supplement: Supplementary file 1 [file Data_Sheet_1.pdf]

Supplementary Material

1 VARIABILITY ANALYSIS

Since the full circuit is analog, a natural question to ask is what happens if components of the circuit have variations. In this section a variational analysis is performed by varying the parameters of the BSN as well as the parameters in the learning part of the circuit.

First, in Fig. S1 the diameter of the circular magnet of the BSN is varied and the learning of a FA performed (compare Fig. 3 in the main manuscript). The diameter of magnet in the BSNs is sampled from the normal distribution

$$N(x) = \frac{1}{\sigma\sqrt{2\pi}} \exp\left[-\frac{1}{2}\left(\frac{x-\mu}{\sigma}\right)^2\right] \quad \text{(S1)}$$

where the mean is given by $\mu = \mu_D = 22$ nm which is the diameter used in the main manuscript. The standard deviation of the diameter σ_D is varied to get a performance measure for different degrees of variation. Fig. S1 shows that variations with σ_D up to 5 nm are tolerable which is about 25% of the overall diameter. That shows that the circuit is robust to variations.

In Fig. S2 the learning rate of every weight in the FA circuit is varied by allowing variation of the resistance value R. For every RC-circuit the value of R is sampled from the normal distribution with the standard deviation of σ_R and a mean resistance value of $\mu_R = 5\ \text{k}\Omega$ which is the resistance value used in the main manuscript. Fig. S2 shows that variation of the learning rate can be tolerated. Interestingly, Fig. S2 also shows that part of the learning curves with high variation show less error than the ideal learning curve where $\sigma_R = 0$. This can be explained by the fact that for some weights, a smaller R results in a faster learning rate that makes the learning converge faster. In addition, the circuit can also have the ability

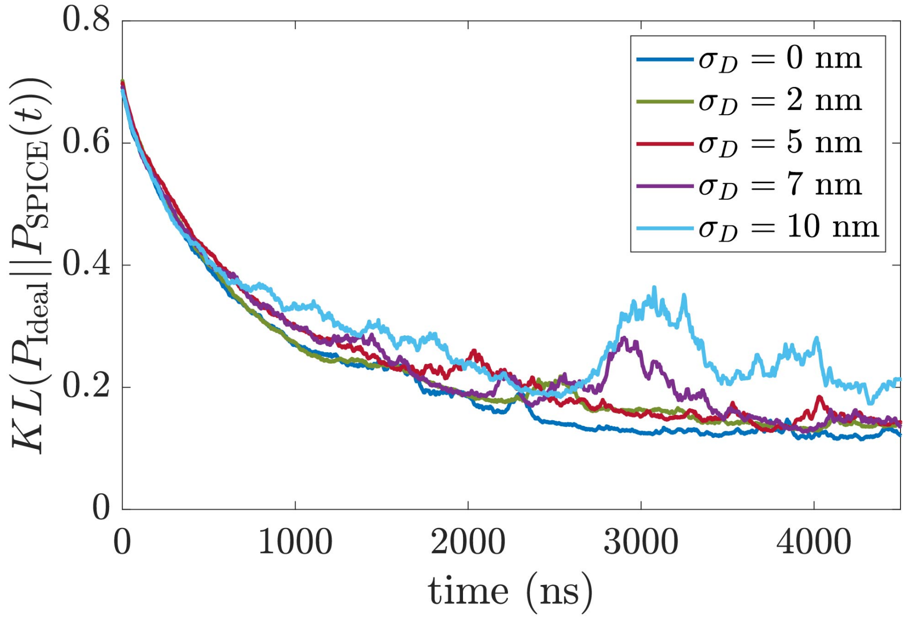

Figure S1. Learning performance of a full adder with 5 p-bits. The diameter of the BSN is varied by sampling from the normal distribution $N(x)$ for different standard deviations σ_D with a mean of $\mu_D = 22$ nm.

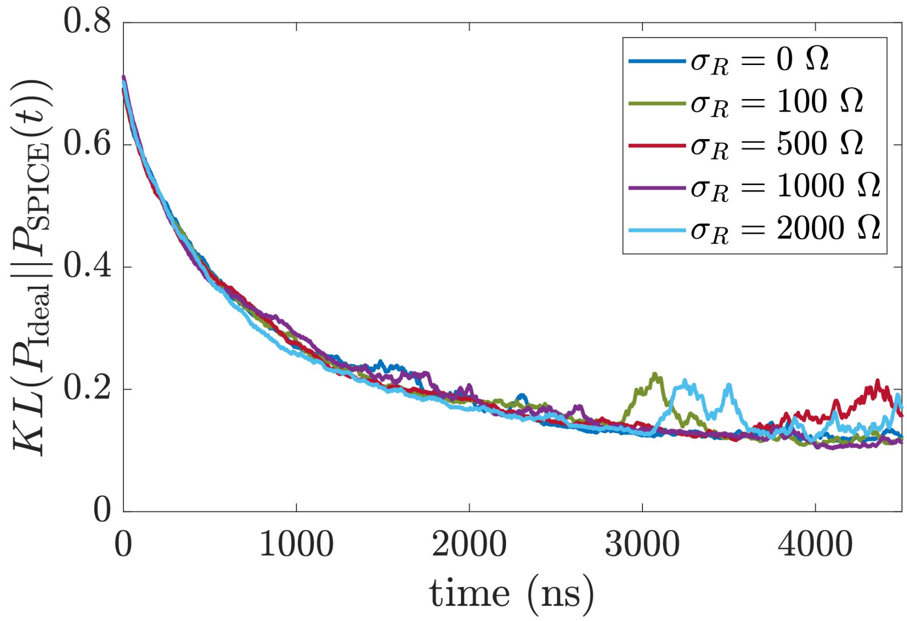

Figure S2. Learning performance of a full adder with 5 p-bits. The resistance of the learning circuit R is sampled from the normal distribution $N(x)$ for different standard deviations σ_R and a mean of $\mu_R = 5\ \mathrm{k}\Omega$.

to learn around varitions. However, increasing the learning rate of all weights can be detrimental to the learning performance since it can prevent the learning from converging.
